# SylvCiT - An AI-based support to urban forest resilience

**DOI:** 10.1371/journal.pone.0339173

**Published:** 2026-02-04

**Authors:** Maxime Nicol, Annick St-Denis, Raouf Moncef Belbahar, Fanny Maure, Arcady Gascon-Afriat, Christian Messier, Marie-Jean Meurs

**Affiliations:** 1 Centre interuniversitaire de recherche sur la science et la technologie (CIRST), Université du Québec à Montréal, Montréal, Québec, Canada; 2 Département des Sciences biologiques, Centre d’étude sur la forêt, Université du Québec à Montréal, Montréal, Québec, Canada; 3 Département des Sciences naturelles, Institut des sciences de la Forêt tempérée, Université du Québec en Outaouais (UQO), Ripon, Québec, Canada; USDA Forest Service Southern Research Station, UNITED STATES OF AMERICA

## Abstract

Urban trees are attracting increasing interest due to their contribution to mitigating some negative urbanization effects. Indeed, trees provide numerous ecosystem services such as carbon sequestration, heat island mitigation, habitats for myriad living creatures, and aesthetic values. However, a lack of tree diversity at the street and neighborhood levels threatens their resilience and service delivery. This article presents SylvCiT, a machine learning and optimization-based system that recommends a diversity of suitable tree species based on functional traits, planting location, and neighboring trees, and therefore maximizes functional diversity at different spatial scales. Special emphasis is placed on human-machine interfaces, including factors that affect user experience, recommendation acceptance and transparency. We show two use cases within SylvCiT. First, we analyze the urban forest of a Montreal neighborhood (Quebec, Canada) in terms of tree diversity, structure, and carbon storage. Second, we assessed species and functional group richness and diversity in 10 parks of Montreal and simulated the effects of planting the recommended species, which resulted in higher species and functional group diversity.

## Introduction

Urban populations are growing rapidly, with over 55% of the world population living in cities and an expected increase to 68% by 2050 [1]. This urbanization leads to issues such as air quality degradation [2], heat islands, and flooding risks [3,4]. Sustainable urbanization strategies are crucial [1]. Thus, urban trees are gaining attention for their role in restoring ecosystems and mitigating urbanization effects, but tree planting programs can have a greater or lesser impact on air quality, carbon sequestration and temperature depending on the species chosen, their size, and their abundance [5–7].

Misunderstanding the mechanisms and dynamics of the delivery of ecosystem services can minimize the impact of tree planting programs [5,6]. In addition, urban trees are increasingly exposed to various threats such as climate change, insect invasions, and exotic diseases due to increasing commercial trade [8–10]. Diversification of tree populations is becoming of paramount importance [11]. Strategic management of urban forests would ensure that urban ecosystems are resilient, i.e., able to absorb or tolerate future disturbances, and therefore continue to provide their functions and services. However, the ecosystem services delivered by a tree or a set of trees vary considerably depending on the environmental and socio-economic characteristics of each planting site, as well as on the characteristics of the tree itself (species, size, and condition).

In this regard, several methodologies [12,13] have been developed and applied in an effort to understand how to manage urban forests to produce more ecosystem services. One of the approaches highlights optimal planting locations, based on ecosystem services, as well as economic and ecological parameters with the goal of mitigating air pollution and urban heat islands [5]. The well-known software suite of tools i-Tree [14], designed by the *USDA Forest Service*, provides urban forestry analysis and benefits assessment tools, but most of them are not spatially explicit and, while the applications themselves are in the public domain and freely available, they are not open source, meaning their source code is not publicly accessible [15].

The main purpose of our research is to design and implement an open-source solution to visualize the geographical distribution of trees, spatially explicit assess urban forests in terms of diversity, structure, and ecosystem services, and finally generate recommendations for planting the most appropriate tree species and functional groups in a given area to strengthen resilience. These recommendations consider neighboring trees to increase diversity and minimize the risks of major tree disturbances on a local scale.

Our work was carried out in a largely multidisciplinary context combining urban forestry, functional ecology, and computer science, and relies on Artificial Intelligence (AI) based approaches. We are using a functional approach based on the biological characteristics of trees (also called functional traits), such as seed mass and wood density, because they are linked to the functions and vulnerabilities of trees and the services they provide [11].

The objectives of this manuscript are: (1) Provide some background on recommender systems and the quantification of ecosystem services; (2) Present and describe the SylvCiT tool, first discussing the calculation of ecosystem services and ecological indices, then describing the implementation of the recommender system and the technologies used for the development of the tool; and (3) Present two use cases of SylvCiT. The final section provides an overview of the project, its challenges and future perspectives. This paper complements our previous publication *An urban forest diversification software to improve resilience to global change* [15].

## Related work

### Recommender systems

Recommender systems (*RS*) are playing an increasingly important role in online applications handling large datasets and curating outputs based on user preferences [16]. These systems draw upon diverse fields such as information retrieval, human-computer interaction, and artificial intelligence. Their applications range from helping users discover items of interest to supporting decision-making processes [17,18].

The work of Melville and Sindhwani [19] identifies three main categories of recommender systems : (1) collaborative filtering, (2) content-based filtering and (3) hybrid approaches. Collaborative filtering employs user interaction data to recommend items based on similarities among them, while content-based filtering relies on the attributes of the items themselves to propose choices [20]. Hybrid approaches aim to address the limitations of each individual method, including cold start issues, sparse matrices, and reliance on detailed metadata [21,22]. Since SylvCiT aims to recommend species based on ecological factors, we have opted for content-based filtering.

Recent works highlight the importance of moving beyond using only accuracy as a performance criterion. Studies like McNee et al. [23] and Ge et al. [24] emphasize the need to incorporate transparency and user experience into recommender systems. The work of Adomavicius and Tuzhilin [25] also advocates for contextualized recommendations and greater flexibility.

Notably, interactive recommender systems have emerged as a promising direction. Parra et al. [26] introduced interfaces that allow users to adjust the importance of different recommendation criteria, enhancing transparency and controllability. Similarly, He et al. [27] proposed integrating visualization techniques into recommender systems to better support user feedback and understanding. Valdez et al. [28] argued for adaptations tailored to diverse user needs and contexts, emphasizing the role of interaction in fostering trust. These works provide valuable insights into the design of recommender systems and have guided the establishment of SylvCiT core principles and priorities : user engagement, decision support, transparency, interactivity and contextual adaptation.

### Quantifying ecosystem services

Recognition of ecosystem benefits has motivated green infrastructure decision makers to implement ambitious tree planting programs [29]. However, while ambitious planting programs are laudable, their success is highly dependent on the careful selection of species adapted to their planting sites and less vulnerable to pests and pathogens [30]. With this in mind, several recent studies provided assessment and decision-support methodologies and tools for urban forest managers to improve the planning, design, and management of urban forests.

The Haase et al. [31] study provided an introduction to the valuation of ecosystem services (ES) in urban areas, the types of ES most commonly studied, and the models most commonly used for their quantification and valuation. The conclusion of their work highlights (in a non-exhaustive way) the scarcity of studies dealing with the temporal and spatial dynamics of ecosystem services despite their importance for urban planning. Furthermore, there is a lack of studies concerning trade-offs and synergies between different ES. Finally, the authors conclude that a spatially explicit assessment of ES at a relatively high resolution (neighborhood and street level) would be essential to integrate the assessments and conclusions of the various studies into urban planning and management strategies.

The literature review by Lin et al. [32] shows the growing interest in the research community in quantifying ecosystem services. The authors identified 242 works and 476 case studies for the period between 1996 and 2017 with more than half of the studies published between 2012 and 2017. Two broad categories of numerical models are currently used for urban forest analysis: general purpose models and urban forest specific models, such as the aforementioned i-Tree suite.

In addition, Lin et al. [32] presented other widely used models. For example, ENVI-met [33] allows the modeling and comparison of designed or real landscape scenarios (e.g., with/without trees, tree and building layout). Computational Fluid Dynamics models (CFD) [34], which are models based on the fundamental laws of fluid mechanics and thermodynamics, allow the users to quantify the thermal effects of trees on surrounding buildings.

ENVI-met and CFD require new parameters related to the study site when applied outside their original modeling domains whereas i-Tree can be used in new locations or conditions without recalibrating the model parameters. Also, one of the limitations of CFD is the need for the user to have programming knowledge.

Regarding statistical models, again according to Lin et al. [32], these are often focused on economics, such as estimating the impact of urban trees on real estate values and energy conservation. It should also be noted that the majority of papers implementing statistical models employ hedonic regression models to estimate, for example, the contribution of ecosystem services to property values.

This summary of various academic works indicates that to date SylvCiT is the only free and open-source recommender system that allows spatially explicit analyses for the purpose of optimizing urban forest resilience. It can assess tree diversity, carbon storage, and ornamental value at multiple scales (e.g. in a park or a neighborhood), and then propose functional groups and species to plant to increase diversity. In the next section, we will discuss the methodology we used to develop this tool.

## Materials and methods

### Data

#### Urban tree dataset.

The urban tree dataset used for the experiments is the inventory of urban trees in the city of Montreal, collected and updated by city inspectors in each borough and available in csv format (*comma-separated values*) on the city’s open data portal [35]. This inventory contains data on municipally owned trees: street and off-street trees (parks and public squares). It lists 313,049 trees (as of August 14, 2024) and includes many variables about each tree, such as species, diameter at breast height (DBH), date of measurement, date of planting, and geographic location expressed in latitude and longitude.

## SylvCiT

In this section, we describe the methodology used to develop SylvCiT. We begin by describing the calculation of ecosystem services (e.g., carbon storage) and ecological indices (e.g., richness, diversity). Next, we discuss the method of improvement calculation. Then, we discuss the approach to tree species recommendation. Finally, we conclude this section by detailing the implementation and the technologies used for the development of SylvCiT.

### Ecosystem services and ecological indices

#### Carbon storage and monetary value.

Lambert et al. [36] developed a system of equations to separate biomass into compartments (wood, bark, branches) based on DBH measurements only, or on DBH and height measurements. These formulas allow us to estimate the aboveground biomass for 33 forest species, and biomass for hardwoods and softwoods. Thus, for our study, we chose to base our calculations on these equations:

$$y_{wood}=\beta_{wood1}D^{\beta_{wood2}}+e_{wood}\;y_{bark}=\beta_{bark1}D^{\beta_{bark2}}+e_{bark}\;y_{branches}=\beta_{branches1}D^{\beta_{branches2}}+e_{branches}\;y_{aboveground}={\hat{y}}_{wood}+{\hat{y}}_{bark}+{\hat{y}}_{branches}+e_{total}$$ (1)

where *y*_*i*_ is the dry biomass (kg) compartment *i* of a living tree (wood, bark, branches, aboveground); ${\hat{y}}_{i}$ is the prediction of *y*_*i*_; *D* is the DBH of the tree (cm); $\beta_{jk}$ are model parameters with coefficient estimates where *j* is wood, bark, and branches; *k* = 1 or 2; and *e*_*i*_ is the error term.

The values of $\beta_{jk}$ are taken directly from Lambert et al. [36]. For species absent from this study, we use the groups of ‘hardwood’ or ‘softwood’ values.

For our work, we chose to ignore the error terms (acting as if each error term was zero). We simply provide estimated values without specifying the margin of error for each unique value to simplify the calculations and make them faster.

Once the above-ground biomass is estimated, we calculate the root biomass. For this, we rely on the work of Li et al. [37] who assumes that the total root biomass (dependent variable) can be estimated from the total above-ground biomass (independent variable). Thus, we use the following two equations:

$$RB_{s}=0.222AB_{s}\;RB_{h}=1.576\times AB_{h}^{0.615}$$ (2)

where *RB* and *AB* are the root and aerial biomass, respectively, and the indices *s* and *h* designate the species type, coniferous (*Softwood*) and deciduous (*Hardwood*), respectively.

The above-ground biomass and the root biomass are summed to calculate the total biomass. However, according to Nowak et al. [38], trees grown in the open tend to have less biomass than predicted by forest-derived biomass equations for trees of the same DBH. To account for this difference, the total tree biomass results are multiplied by a factor of 0.8 [39]. Thus, the total biomass for a tree is obtained by:

$$biomass_{total}=y_{total}+RB\;biomass_{urban}=0.8\times biomass_{total}$$ (3)

For the calculation of carbon storage, total biomass is simply multiplied by 0.5 [40].

Finally, to estimate the monetary value associated with carbon storage by urban trees, the carbon values were multiplied by $994.57 (2025 CAD) per ton of carbon, corresponding to the social cost of carbon, a measure that estimates damages from climate change impacts [41].

#### The functional approach.

Until the last decade, the choice of tree species and their location in cities was motivated principally by aesthetic criteria, acceptability to the population, tree supply, and tolerance of certain stresses encountered in urban environments [42–44]. This strategy is problematic because only a few species end up being used repeatedly and make up most trees in the city [45]. In addition, there is a high degree of similarity between the characteristics (functional traits) of these trees, resulting in lower tree resilience and greater sensitivity to similar threats [46].

Functional traits represent morphological, physiological, or phenological characteristics of an organism that affect its individual performance in terms of ecosystem service delivery and determine its response to one or more environmental factors [47]. Functional traits include but are not limited to specific leaf area, wood density, as well as flood and drought tolerance. The greater the functional diversity, the less likely a large proportion of the tree community will be negatively affected by a certain disturbance. This quantification allows the user to reflect the diversity for a street or neighborhood and also to better define diversification objectives and to quantify the changes to be made to reach them [11].

Another technique based on functional diversity is the use of functional groups [11,48]. The groups that we are using are based on seven functional traits: seed size, specific leaf area, leaf nitrogen content, wood density, shade tolerance, drought tolerance and flood tolerance [49]. The functional trait values for each species were obtained from the Tree Trait Task Force (3TF) - Tree Functional Trait Database [50], which integrates data from an extensive literature review and the TRY plant trait database. Species dissimilarity based on these trait values was assessed using hierarchical clustering methods, allowing species with similar functional profiles to be grouped together [49]. The resulting classification into functional groups is presented in Table 1.

**Table 1:** The ten functional groups and representative species for each group, based on the method presented in [11] and on the groups presented in [49].

Functional Group	Description	Representative species (Scientific name)	Representative species (Common name)
1A	Conifers generally tolerant to shade, but not to drought or flooding.	*Picea, Abies, Thuja spp., Pinus strobus*	Spruce, fir, cedar and white pine
1B	Drought tolerant, but intolerant to shade conifers.	*Pinus, Larix, Juniperus, Ginkgo spp.*	Pines, larches, junipers and ginkgo
2A	Shade-tolerant trees with broad, thin leaves and medium growth.	*Acer, Tilia, Magnolia, Fagus spp.*	Most maples, basswood, magnolia, beech
2B	Resembles 2A except for the very heavy seeds dispersed by gravity.	*Aesculus spp.*	Chestnut trees
2C	Large flood tolerant trees.	*Ulmus, Fraxinus spp., Celtis occidentalis, Acer rubrum, Acer saccharinum, Acer negundo*	Most elms, ash, hackberry, red maple, silver maple and Manitoba maple
3A	Small drought tolerant trees, heavy wood, thick leaves, low growth.	*Rosaceae spp. (Sorbus, Pyrus, Crataegus, Amelanchier spp.), Syringa spp.*	Rosaceae species (Mountain ash, pear tree, hawthorn and serviceberry) and lilacs
3B	Medium group. Intolerant to flooding.	*Rosaceae spp. (Prunus, Malus spp.), Catalpa, Maacka spp.*	Large Rosaceae (cherry tree, apple tree), Catalpa, Maackia and other miscellaneous species
4A	Large trees with heavy seeds and wood. Drought tolerant.	*Quercus, Juglans, Carya spp.*	Oaks, walnuts, and hickories.
4B	High tolerance to drought, but not to shade or flooding. Heavy seeds, rich leaves.	*Fabaceae spp. (Gleditsia triacanthos, Robinia pseudoacacia, Gymnocladus dioicus)*	Legumes (honey locust, Kentucky coffeetree, black locust)
5	Pioneer species with very small seeds. Fast growing, flood tolerant, light wood.	*Populus, Salix, Betula (*except *Betula alleghaniensis), Alnus spp.*	All poplars, willows, alders and birches (except yellow birch)

Species are classified as two groups of conifers (1A, tolerant to shade, but intolerant to drought, 1B, intolerant to shade, but tolerant to drought), and eight groups of broadleaved species: tall species (2A, 2B, 2C), small and medium species (3A, 3B), large-seeded species (4A, 4B), and pioneer species (5).

These functional groups are intended to help managers and citizens to increase urban forest resilience by planting tree species with different traits and different responses to threats [11]. Thus, we decided to build our recommendation approach on suggesting functional groups to users rather than simply a list of tree species. The abundance of trees per functional group is used to estimate the functional diversity.

#### Richness and diversity.

Richness refers to the number of species or the number of functional groups in a given sample, community or area. Diversity takes into account the relative abundance of trees by species or by functional group. To assess diversity, the effective number of species (ENS) and the effective number of functional groups (ENFG) are calculated, based on the exponent of the Shannon Index. An ENFG of 1 means that the diversity is very low since only one functional group is present, while an ENFG of 10 (maximum value) means that the diversity of the functional groups is very high since all the groups are present and the trees are evenly distributed among the 10 groups (Table 2).

**Table 2:** Levels of functional diversity based on the effective number of functional groups (ENFG).

ENFG	Diversity Level
1 to 3	Very low
3 to 5	Low
5 to 8	Intermediate
8 to 10	High

#### 10-20-30 Rule (Santamour).

The 10-20-30 rule, also known as the Santamour rule, is a guideline for reducing the risk of catastrophic tree loss. The rule suggests that an urban tree population should not include more than 10% of a species, 20% of a genus, or 30% of a family [51]. The 5-10-15 rule is also increasingly used as a quick estimate of the diversity of an urban forest [52]. The tree inventory should not contain more than 5% of the same species, 10% of the same genus and 15% of the same family. These two rules of thumb are presented in SylvCiT in a graphical manner with thresholds for each species, genus and family.

### Improvement calculation

To calculate the improvement made by adding a tree of a specific species to the inventory, we use the following formula:

$$\frac{n_{1}}{n_{0}}-1=m$$ (4)

where *n*_0_ is the effective number of species before the addition of a new tree,

*n*_1_ is the effective number of species after the addition of a new tree and
*m* is the rate of improvement.

Note that we have to calculate the effective number of species twice: once before adding the tree and then after adding the tree. The same method is used for the calculation of the improvement of ecosystem services or for the calculation of the improvement of other ecological indices when a new tree is added.

### Recommendation algorithm

We hypothesize that a visual interface that gives users control over the recommendation process with a good visualization would improve the user’s experience and enhance their opinion about the quality of the recommendations.

In designing SylvCiT, we analyzed existing interactive recommendation systems and identified multiple visualization techniques used to support user control by improving or exploring recommendations. Among these, sliders and graphics with moveable elements were recurrent features. We therefore adopted these elements which, among other things, improve the user’s interaction with the system and give them full control over the recommendations according to their criteria.

We propose a tight integration of visualization and recommendation techniques to allow users to interact with the recommender system at each step. For instance, we provide them with the possibility to weight the input data and the recommendation criteria according to their objectives.

In the case of SylvCiT, the main objective is to optimize the resilience and increase the ecosystem service productivity of an urban forest by increasing the functional diversity. Thus, increasing functional diversity is the main factor and it can not be removed by the user. The user’s objectives can be an input to the system and can be defined as prioritizing one ecosystem service over another (e.g., increasing carbon storage). In these terms, the objective of the system is to identify the most suitable tree species, combine them with the existing urban forest, and recommend the most favorable species and functional groups to be planted.

A description of the urban forest includes the following information:

- The geographic location of the trees being analyzed;
- The species of these trees;
- The functional groups of each tree in the selection.

The user’s goals are used to refine the recommendation results and rank them.

Thus, the recommendations of SylvCiT:

- Are addressed in the right geographical and climatological context;
- Are personalized, as they target the objectives defined by the user;
- Serve a specific purpose, which is to functionally diversify the urban forest.

Based on the trees selected by the user and after they specify the importance of each index to be improved, the database of candidate tree species is reduced to only the tree species which meet the 10-20-30 rule [53]. This is because an inventory that meets the 10-20-30 rule is considered more diverse than one that does not [54]. Note that the candidate tree species database includes both native and ornamental species, but excludes invasive species (*e.g., Rhamnus* spp.), following the recommendations published by the government of Quebec [55], as well as species severely affected by pest and disease (*e.g., Fraxinus* spp., *Juglans cinerea*). These excluded species were selected in collaboration with urban forestry experts from our municipal partners.

Next, we calculate the improvement that a new tree would bring to a given area. Then, we weight the improvement to each index by the value provided by the user, sum the weighted values, and finally calculate a score for each species.

Finally, a list of species sorted by score is recommended. This list should be used to make decisions about the most appropriate species for an area given the desired tree characteristics. The score is calculated using the following formula:

$$Score=\sum_{i=1}^{5}w_{i}x_{i}$$ (5)

where

- *i* indexes the improvement criteria considered:
  1. Functional group diversity,
  2. Functional group richness,
  3. Species diversity,
  4. Species richness,
  5. Carbon storage.
- *w*_*i*_ is the weight applied to each index *i*:
  - For functional group diversity, and functional group richness (i∈{1,2}), $w_{i}\text{is fixed at}10$.
  - For species diversity, species richness, and carbon storage (i∈{3,4,5}), *w*_*i*_ is user-defined, where $w_{i}\in [1,10]$.
- *x*_*i*_ is the improvement value on an index achieved by adding a tree to the inventory.

### SylvCiT: Implementation and technologies

An effective recommendation algorithm is essential to inspire confidence in a recommender system. However, the interfaces and the technologies implemented play roles as the quality of the recommendations [56].

Our approach is based on three steps:

1. Inventory and assessment of urban forest composition in terms of diversity, structure and ecosystem services delivered;
2. Recommendation of new tree species to be planted according to the needs specified by the user in terms of number, but also in terms of service to be prioritized;
3. Simulation of the services delivered by the set of trees analyzed with the addition of the recommended trees to evaluate the contributions to the ecosystem services delivered.

SylvCiT was implemented using a variety of frameworks and libraries best suited to each of the necessary tasks. The graphical user interface was designed using mockup builder Balsamiq [57]. The interface was designed according to established usability guidelines [58,59], with the aim of maximizing usability for non-initiated users.

Not only do we intend to help researchers and less qualified persons in the field to make their analyses with ease and confidence, but our tool also follows the 20 simple rules that have been released for making software robust enough to be run by anyone, anywhere with a good usability [58,59]. The updates as well as the releases of the source code are realized by means of a GitLab repository. Users are aided in getting started quickly by extensive documentation and examples.

**Fig 1:**
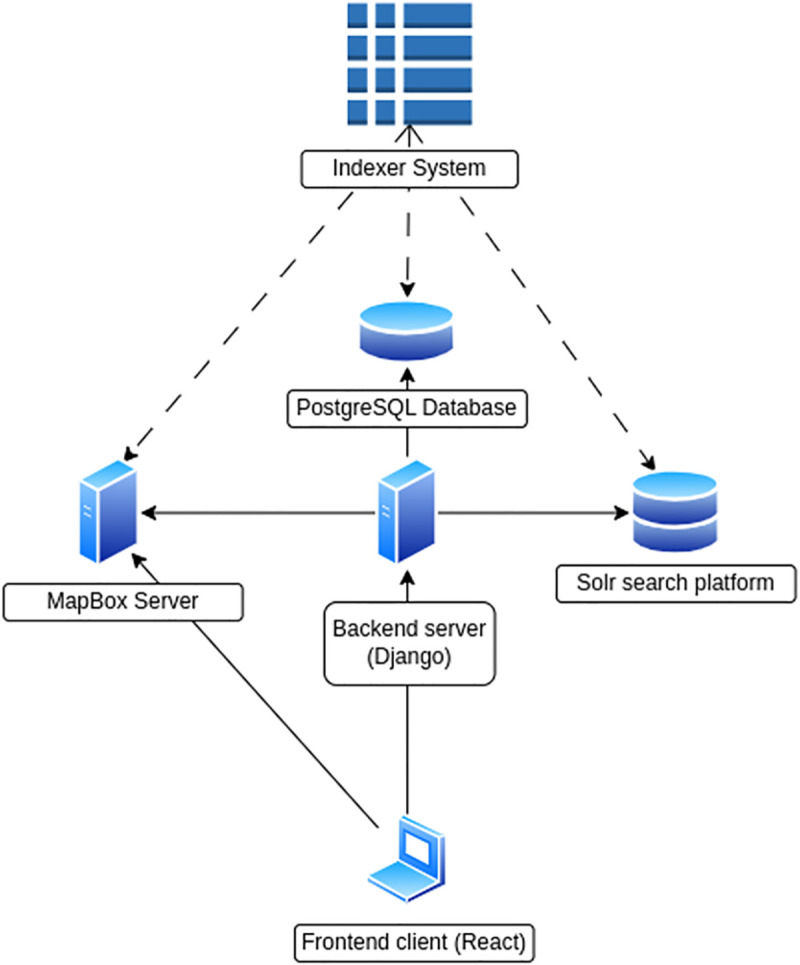
System architecture - SylvCiT.

The system currently runs on a Linux server, but it can run on a wide variety of platforms thanks to Docker [60]. Our tool includes only open-source technologies and is based on Apache Solr [61], which is itself an open-source enterprise-search platform based on the Apache Lucene [62] indexing engine. Solr is designed for scalability and fault tolerance. This design enables real-time indexing capabilities, as well as high-speed query processing. The backend application was built using Python, an open-source scripting language, and Django [63], a Python-based web framework that utilizes MySQL as the database engine. The front-end application was developed using ReactJS [64], a popular framework for creating web-based user interfaces. In addition, we utilize MapBox, a free map data hosting service, to render all geolocation images. To ensure comprehensive inventory data, we employ an indexer system capable of integrating data from various sources into a unified format (Fig 1).

The source code is publicly accessible and available for easy replication of results. The system is published under the GPL-v3 license. It is available in the following repository:

https://gitlab.com/ikb-lab/ikb-lab1/data-science/sylvcit (Accessed 2025-12-06). To allow users to independently manipulate SylvCiT and to visualize the public trees of Montreal, to perform analyses and to receive recommendations, we have made the tool publicly available at the following address : https://sylvcit.ca/ (Accessed 2025-12-06).

In terms of continuous development and release, SylvCiT follows a punctual update cycle to ensure alignment with both current scientific knowledge and urban tree inventories. The system undergoes at least a yearly update process for values such as the social cost of carbon, DBH percentiles, and species within functional groups. Furthermore, the urban tree inventories are updated whenever a municipal partner or public entity releases a new dataset. Both types of revisions are validated by our multidisciplinary research team before integration, ensuring that SylvCiT remains scientifically accurate and consistent with current urban landscapes.

## Results and discussions

### SylvCiT

#### Use case #1 - Neighborhood of Montreal.

We developed a plausible use case of SylvCiT for planting new trees. The objectives were to demonstrate what the tool is capable of and how it helps to better understand the urban forest and to provide decision support for the selection of the best tree species to be planted towards maximizing functional diversity and, consequently, improving resilience of the urban canopy.

Since an urban forest study is usually done for a neighborhood or borough, we selected Verdun, a 970 hectare neighborhood of Montreal where about 70,000 people live [65]. We chose Verdun specifically because we feel it represents the average urban neighborhood in terms of volume of trees and diversity. The overall goal was to undertake a realistic assessment of the carbon storage potential of the Verdun neighborhood. The specific objectives of the study were as follows:

- Characterize a comprehensive inventory of trees in the neighborhood with an accurate measurement of species richness and functional diversity;
- Quantify the gross carbon storage of the urban forest and its monetary value;
- Assess the impact of planting the suggested tree species.

As a starting point, SylvCiT home interface allows the user to visualize the distribution of trees on a map. The color and diameter of each point on the map indicate the species and the DBH, respectively. In addition, a list of the proportion of trees by species in the current view provides an overview of the most abundant species.

In our specific use case, it was easy to recognize that ash and maple (*Fraxinus* and *Acer*, respectively) represented more than 30% of the urban forest selected; these species are an important part of Montreal’s tree heritage in general. Other statistics are also available, such as the number of trees in the view and the number of species.

Furthermore, when clicking on a tree in the map, specific details about the tree are displayed including species, family, genus, functional group, current DBH, date of planting and date of measurement (if available), and the maximum DBH. The maximum diameter at breast height (max DBH) is taken as a proxy to identify old trees [66]. The trees in each species across 25 combined tree inventories of southern Ontario and Quebec cities are ranked into DBH percentiles as an aggregated species grouping. For each species cohort (with a minimum of 150 trees), those within the 95th percentile were described as the max DBH. For species with less than 150 trees, we calculated the average data for the species of the same genus or hybrid.

The user has the option to change the displayed layer, for example, to a layer that visualizes the heat islands in the studied area. Finally, this interface allows the user to select a set of trees for analysis. In our case, we have selected an area of about 335 hectares, with 4244 trees.

**Fig 2:**
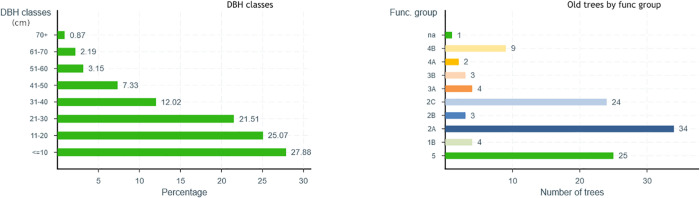
Proportion of species by DBH class and distribution of old trees by functional group (see Table 1) - SylvCiT.

During the analysis, information about species richness and diversity as well as functional group richness and diversity is calculated based on the selection made. Fig 2 shows the distribution of the trees according to their DBH class as well as their functional group. This indicates that the tree cover of Verdun is more or less young, with more than half of the trees not exceeding a DBH of 20 cm, which will eventually ensure the succession of mature trees. However, it should be noted that special attention must be paid to young trees to ensure their survival. This significant presence of young trees is the result of the arboricultural plan undertaken by the borough of Verdun in 2014 to increase its forest cover as well as the felling operations of ash trees suffering or likely to suffer from the emerald ash borer [67]. In the graph presenting the proportion of old trees by functional group, we note the abundance of trees from groups 2A and 2C, which are mostly maple (*Acer* spp.), elm (*Ulmus* spp.) and ash (*Fraxinus* spp.), representing the species historically planted in Montreal. These groups represent 32.83% of the trees for 2A and 20.55% for 2C.

SylvCiT also provides an estimate of carbon storage and its monetary value, related to the social carbon cost. For the tree set in our selection, carbon storage is 916,462.55 kg for a value of 911,486 CAD (at a value of 994.57 CAD per ton of carbon in 2025) [41]. These numbers can be explained by the relative young age of the urban forest analyzed.

**Fig 3:**
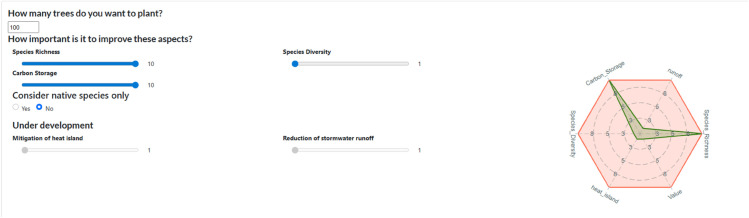
Choice of the number of trees and weighting criteria - SylvCiT.

Fig 3 shows how the user can choose the number of trees to plant and weight certain recommendation criteria according to their needs. For this scenario, we chose to improve species richness and carbon storage potential.

**Fig 4:**
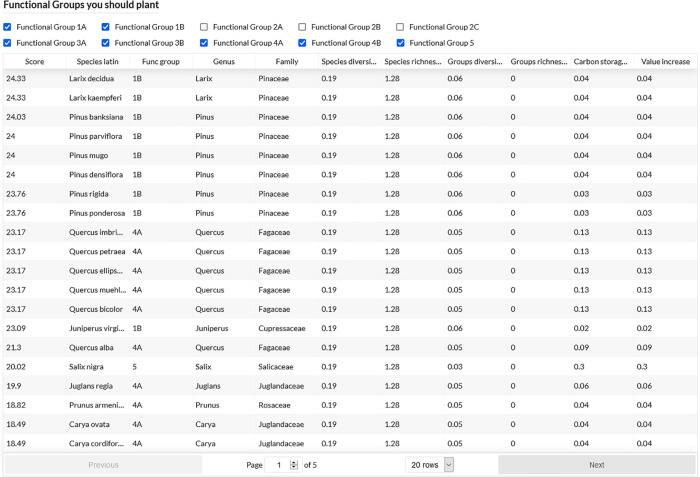
List of recommended groups and species - SylvCiT.

Fig 4 provides an overview of the recommended functional groups/species based on the tree selection and criteria chosen in the previous step. The recommendation of the trees is in a decreasing order according to the score calculated according to the improvement brought by the species on diversity, richness and carbon storage. Each criterion is weighted according to the choices made in the previous interface. The user can choose to remove or include functional groups from which species will be recommended. It is noticeable that the functional groups that are least present in the analyzed set are the most recommended. An interesting fact to note is that group 2B was not recommended, despite its minimal presence in the analyzed set. This could be explained by the fact that among our candidate species for recommendation, only 13 species belong to group 2B, consisting of chestnut (*Aesculus* spp.) trees. Interestingly, species in group 1B (comprising only 103 trees out of 4244 analyzed) scored the highest and represented 17.2 % of the recommended species. Group 4A represents 12.9% of the recommended species.

As the objective of our approach is to increase functional diversity, in the following section we developed another use case applied to the parks of Montreal to estimate the capacity of the tool to recommend trees of functional groups absent within a selection or to balance the distribution of groups.

#### Use case #2 - City parks in Montreal.

We wanted to assess the impact of the recommended species on the functional diversity in 10 parks in Montreal containing a variable number of trees. The parks and the number of trees are detailed in Table 3. This case is a concrete example of a new tree planting operation. Thus, for our case study, we chose to plant 5% of new trees compared to the number of trees already planted.

**Table 3:** Selected parks, number of trees already present in the park and number of trees to plant.

Park name	Number of trees	Number of trees to plant
Beaubien	257	13
Maisonneuve	6329	316
Jarry	2299	115
Gabriel-Lalemant	176	9
Delorme	1025	51
Ahuntsic	944	47
La Fontaine	2862	143
Lacoursière	346	17
Angrignon	935	47
Jeanne-Mance	407	20

In the continuity, we simulated a choice of trees among the recommended trees by filtering the list with the help of the 10-20-30 rule, which states that we should plant no more than 10% of trees of the same species, no more than 20% of the same genus and no more than 30% of the same family [51].

This rule assures us of making a choice of trees to be planted that would respect our initial goal of improving diversity. Then, SylvCiT recommends species that contribute to maximize the functional group diversity. Once the filtering was done, we chose the top 10 recommended species. We thus plant each species with one-tenth of the total number of trees defined earlier (Table 3).

Table 4 shows the improvements in 10 parks of Montreal following planting of the recommended species. For this analysis, we focused on improving the diversity. Even though all groups were present in 9 out of 10 analyzed parks, we can see an average improvement in species richness across all the parks analyzed of 18.2%, an average improvement in species diversity of 15.9%, and finally an average improvement in functional group diversity of 8.5%. Also, it should be noted that only Jeanne-Mance park had 8 functional groups present out of 10, so SylvCiT recommended the species of the missing functional groups.

**Table 4:** Species richness and diversity improvements (a) and functional group richness and diversity improvements (b) occurring in parks after planting recommended species.

a)						
**Park name**	**Current status**		**Status after SylvCiT recommendations**			
	**Species richness**	**Species diversity**	**Species richness**	**Species diversity**	**Species richness improvement** (%)	**Species diversity improvement** (%)
Beaubien	40	24.9	50	28.2	25.0	13.3
Maisonneuve	81	29.1	89	33.2	9.9	14.1
Jarry	76	32.0	82	35.5	7.9	10.9
Gabriel-Lalemant	47	27.0	56	31.3	19.1	15.9
Delorme	68	26.6	76	30.4	11.8	14.3
Ahuntsic	73	25.9	82	29.9	12.3	15.4
La Fontaine	89	22.6	98	26.1	10.1	15.5
Lacoursière	32	13.5	40	15.8	25.0	17.0
Angrignon	41	15.8	51	18.9	24.4	19.6
Jeanne-Mance	25	4.3	34	5.3	36.0	23.3
**Average**	**57**	**22.2**	**66**	**25.5**	**18.2**	**15.9**
**b)**						
**Park name**	**Current status**		**Status after SylvCiT recommendations**			
	**Functional group richness**	**Functional group diversity**	**Functional group richness**	**Functional group diversity**	**Functional group diversity improvement (%)**	
Beaubien	10	8.7	10	9.0	3.4	
Maisonneuve	10	7.8	10	8.2	5.1	
Jarry	10	8.3	10	8.7	4.8	
Gabriel-Lalemant	10	7.0	10	7.4	5.7	
Delorme	10	7.3	10	7.8	6.8	
Ahuntsic	10	6.4	10	6.9	7.8	
La Fontaine	10	5.5	10	6.0	9.1	
Lacoursière	10	5.0	10	5.6	12.0	
Angrignon	10	4.8	10	5.5	14.6	
Jeanne-Mance	8	2.5	10	2.9	16.0	
**Average**	**10**	**6.3**	**10**	**6.8**	**8.5**	

#### Discussion.

Our study illustrates the importance of analyzing the current state of the urban forest to inform future planning and management decisions. Our recommendations are made based on the current state of the analyzed forest and are part of a comprehensive and integrated tree management strategy at the scale of a neighborhood, borough, or city. In addition, a spatially explicit evaluation of the carbon storage at a relatively high resolution (at neighborhood and street level) is integral to the tool offering better integration into urban planning and management strategies [31].

The SylvCiT database contains more than 220 candidate species for recommendation, selected by the urban forestry team collaborating on the project. As a result, SylvCiT considers a broad spectrum of tree species for various urban contexts. The primary goal of SylvCiT is to optimize the functional group diversity, but it could measure and improve other metrics such as the species diversity and richness and ecosystem services such as carbon storage.

In one use case, SylvCiT recommended species and functional groups in order to increase diversity as a priority, but also carbon storage. Thus, even if many species of groups 2A and 2C, such as maple species, have a high potential of carbon storage, they are not suggested because they are already abundant in the selected urban area.

The other use case focused on parks, which usually contain trees that are more diverse than street trees. Nevertheless, SylvCiT successfully increased the species richness, and the species and functional diversity of the 10 parks tested by adding 5% of new trees for each park. The biggest increase in diversity was for Jeanne-Mance Park, dominated by Silver maple (Acer saccharinum) from group 2C with an increase of 16% in functional diversity and 23.3% in species diversity with only 20 new trees planted.

The increase in functional diversity shown in these two use cases would reduce the risks of having a large proportion of trees damaged by any type of disturbance and help to maintain forest cover over time [11]. In order to reduce the dominance of certain species, genera and families, the recommendation algorithm selects in the first-place species that respect the 10-20-30 rule, a practice that has been frequently adopted in urban forest management plans [54]. We chose this approach instead of the stricter 5-10-15 rule, as in practice it is more difficult to apply due to the limited selection available at nurseries, as well as the cost and uncertainty associated with testing and maintaining new or uncommon species [52].Then, following the recommendation algorithm’s evaluation, species and functional groups that contribute most to functional diversity are recommended to the user.

SylvCiT provides valuable analysis and decision support for tree selection by urban planting decision makers, with the main idea of having the right tree in the right place, and demonstrates that a high functional diversity of trees makes the forest more resilient to different known and unknown stresses. This ensures that our urban forests maintain tree species from many different functional groups, in roughly equal proportions and with some redundancy. Diversification of planted species is also particularly important for the variety of ecosystem services produced as each species provides unique benefits.

In addition to these ecological considerations, SylvCiT is designed to be used by a wide range of stakeholders in urban forestry, from novices to experts. The tool considers how certain characteristics of a recommendable item meet the user’s needs and preferences, the context and usefulness of the item to the user are considered [68]. As we recognize the individual judgment of different groups of users, we refrain from providing strict suggestions for planting species. Finally, the management and planning of urban forests is subject to several constraints, such as the availability of species in nurseries and aesthetic considerations.

#### Limitations.

Even though SylvCiT offers the possibility to enhance urban forest management, it contains limitations that need to be highlighted. Currently, the tool is restricted to the Greater Montreal area (Quebec, Canada) due to data availability and ongoing municipal partnerships. Supporting other regions would require additional collaborations and the integration of new datasets since the tool relies on existing tree inventory and local data such as the social carbon cost. In particular, the definition of functional groups adapted to a different study region and different tree species is essential for the effective use of the tool. The current version of SylvCiT serves as a proof of concept tailored to southwestern Quebec. A more comprehensive database, including a broader range of species, is currently being consolidated to establish functional groups suitable for urban tree inventories in the northeastern North America. For other regions, however, new databases will need to be compiled, and new functional groups defined accordingly.

The reliability of SylvCiT recommendations and ecosystemic maximization is inherently tied to the quality and availability of input data. This means that while the tool is robust for abundant tree species, its performance might be less reliable for scarcer species. Similarly, our carbon storage estimation for trees to be planted assumes a fixed DBH of 15 cm. While this simplification provides a standardized approach that reflects current inventory averages, it does not fully account for varying growth patterns across species and environments. Additionally, the customizations offered to users through manual weighting sliders, while valuable for tailoring the experience to specific urban planning needs, introduce potential biases that may conflict with the default optimization goals described in this article, such as maximizing tree diversity.

Another limitation lies in the current inability of the tool to consider local genetic adaptations of tree species, which could be crucial for urban forest resilience. Furthermore, the tool’s focus on individual tree species selection after the initial functional group recommendations could inadvertently overlook ecosystemic benefits or diversity because of user selection.

Finally, despite ongoing research projects, SylvCiT does not yet include important ecosystemic services, such as mitigation of urban heat islands and reduction of stormwater runoff. The monetary valuation of such services is also not yet available, limiting its applicability in the system.

#### User weighted criteria and manual tree selection.

As previously stated, part of the core principles of SylvCiT is contextual adaptative decision support. This is specifically reflected in the user’s ability to weigh the different criteria considered in the recommendation algorithm, as well as the option to manually select species instead of relying solely on the provided recommendations. This flexibility empowers users to tailor decisions to context-based priorities. While we acknowledge that these customizations might affect the theoretical accuracy of the recommendations, we emphasize that they are a necessity that caters to the pragmatic constraints of urban planners. These may include limited access to certain species of trees, short term urban planning goals, or even budgetary limitations. We also feel it is important to emphasize that while these recommendations will not be as accurate as possible, they are not inherently suboptimal because they reflect hard realities that cannot be overruled in real life.

#### Future perspectives.

Currently, SylvCiT has several limitations, but many research avenues are available to us to resolve them. Urban forestry is a growing field that is interested in the application of computational techniques to solve certain problems encountered by cities. Indeed, there are challenges that we must overcome during the continuing development of SylvCiT, especially with the appearance of new technologies allowing us to accelerate certain treatments or displays.

One of the most important challenges is to implement recommendation algorithms based on machine learning methods. We plan to explore an approach based on reinforcement learning [69], which seems suitable for the nature of the recommendation problem we are dealing with, as this approach creates a system that is constantly learning, does not need periodic updates, and can easily adapt to changes in the characteristics of the analyzed tree sets. However, for training such algorithms, one needs (1) to use large datasets of tree set-based planting recommendation scenarios, or (2) to train these algorithms as the tool is used to take advantage of feedback from the people using it. The latter is our preferred option.

Improving the transparency and controllability of the tool during the recommendation process is another important goal of SylvCiT. The literature on recommender systems advises the support of more advanced levels of interactions, such as controls that define what data can be tracked and considered and for what purposes [28,70]. It is also important to adapt recommender systems and their user interfaces to different personal and contextual characteristics [25,28].

Furthermore, we are working on incorporating many more parameters when making recommendations, including planting and maintenance costs, species preferences for planting site (e.g., streets, parks), as well as abiotic and biotic risks such as flood, drought, diseases, and insect outbreaks. Based on a literature review and a method by Esperon-Rodriguez et al. [71], we are consolidating a list of species for planting in urban heat islands and another adapted to climate change. The tool is currently being tested by our municipal partners and these new lists will be verified by them. Their input will help to validate and improve the recommendations given by SylvCiT. For instance, at their request, we added an option in the tool allowing for native species recommendations.

Reliable models of urban tree growth over time are also a critical component of SylvCiT development. For example, we are developing DBH prediction models for urban tree species common in Montreal as such models have not yet been created. These models are useful for (1) selecting appropriate species for available planting sites; (2) anticipating future tree maintenance, removal costs, and tree replacement; and (3) quantifying the benefits provided by trees [72].

## Conclusion

In this paper, we have proposed a tool which allows a user to analyze the urban forest and recommends tree species well adapted to the existing species composition, to the environment and to user requirements. This tool integrates the calculation of ecosystem services and various ecological indices (e.g., richness, diversity) to provide a quick and simple view of an urban forest at different scales.

Also, we propose a recommendation of tree species adapted to an urban context. These recommendations aim to maximize forest resilience while directly incorporating environmental and economic valuation metrics, such as functional group diversity and carbon storage. By ensuring tree survival and adaptability, the system also enhances the long-term social benefits these trees will provide to the population.

Thus, we have integrated our different experiments into a tool which allows us to visualize the geographical distribution of trees, to quantify the services rendered by each species and finally to generate recommendations for the planting of tree species within functional groups.

This type of system provides a better understanding of the environment around us and has great potential to increase the resilience of our urban forests. Urban planners and managers will be able to quickly analyse their urban forest in a few clicks and at the same time estimate its functional and species diversity without having to perform long analyses or numerous index calculations. In addition, the recommendation module is the first of its kind to guide municipal stakeholders in diversifying their plantations in order to minimize the risks associated with global changes [11,15]. Overall, this project highlights the challenges we may face as well as the opportunities that may arise in the field of urban forestry and artificial intelligence.
